# Rosin Soap Exhibits Virucidal Activity

**DOI:** 10.1128/spectrum.01091-21

**Published:** 2021-12-22

**Authors:** Stephen H. Bell, Derek J. Fairley, Hannele Kettunen, Juhani Vuorenmaa, Juha Orte, Connor G. G. Bamford, John W. McGrath

**Affiliations:** a School of Biological Sciences, Queen’s University Belfast, Belfast, United Kingdom; b Regional Virology Unit, Royal Victoria Hospital, Belfast Trust, Belfast, United Kingdom; c Hankkija Oy, Hyvinkää, Finland; d Forchem Oy, Rauma, Finland; e Wellcome-Wolfson Institute for Experimental Medicine, Queen’s University Belfast, Belfast, United Kingdom; Texas A&M University

**Keywords:** antimicrobial, antiviral, fomite, inactivation, soap, virucidal, virus

## Abstract

Chemical methods of virus inactivation are used routinely to prevent viral transmission in both a personal hygiene capacity but also in at-risk environments like hospitals. Several virucidal products exist, including hand soaps, gels, and surface disinfectants. Resin acids, which can be derived from tall oil, produced from trees, have been shown to exhibit antibacterial activity. However, whether these products or their derivatives have virucidal activity is unknown. Here, we assessed the capacity of rosin soap to inactivate a panel of pathogenic mammalian viruses *in vitro*. We show that rosin soap can inactivate human enveloped viruses: influenza A virus (IAV), respiratory syncytial virus, and severe acute respiratory syndrome coronavirus 2 (SARS-CoV-2). For IAV, rosin soap could provide a 100,000-fold reduction in infectivity. However, rosin soap failed to affect the nonenveloped encephalomyocarditis virus (EMCV). The inhibitory effect of rosin soap against IAV infectivity was dependent on its concentration but not on the incubation time or temperature. In all, we demonstrate a novel chemical inactivation method against enveloped viruses, which could be of use for preventing virus infections in certain settings.

**IMPORTANCE** Viruses remain a significant cause of human disease and death, most notably illustrated through the current coronavirus disease 2019 (COVID-19) pandemic. Control of virus infection continues to pose a significant global health challenge to the human population. Viruses can spread through multiple routes, including via environmental and surface contamination, where viruses can remain infectious for days. Methods for inactivating viruses on such surfaces may help mitigate infection. Here, we present evidence identifying a novel virucidal product, rosin soap, which is produced from tall oil from coniferous trees. Rosin soap was able to rapidly and potently inactivate influenza virus and other enveloped viruses.

## INTRODUCTION

Even before the current pandemic engendered by severe acute respiratory syndrome coronavirus 2 (SARS-CoV-2), the virus that causes coronavirus disease 19 (COVID-19), respiratory-borne viruses were a leading cause of global morbidity and mortality (1). By way of example, viruses such as influenza viruses (which includes influenza A virus [IAV]), are responsible for hundreds of thousands of deaths annually (2). To date, the SARS-CoV-2 pandemic has claimed the lives of over 3.5 million people, and >180 million cases have been reported worldwide (3). Strategies to treat and control the spread of viruses, such as antiviral therapies and vaccines, are employed to protect the health and well-being of the general population in particular for those in at-risk settings, such as in hospital care and in the care sector (4). Pathogenic respiratory viruses may spread directly from person to person via small droplets or aerosols, as well as through direct contact with each other and with contaminated surfaces or fomites (5). Furthermore, aerosolization of environmentally contaminated infectious virus has been observed and can spread disease (6, 7). Infectious SARS-CoV-2 has been shown to persist on surfaces such as metal and plastic for up to 3 days (8). An additional layer of defense against infectious agents like viruses is the destruction of their survival on surfaces.

The infectious particle of many respiratory viruses is encased in a phospholipid bilayer or “envelope,” which is essential for infectivity (9). For infection, enveloped viruses fuse their lipid envelope with the outer membrane, whether that is the plasma membrane or from vesicles, of the target host cell. Enveloped viruses include but are not limited to influenza viruses, CoVs, paramyxoviruses, and pneumoviruses. By comparison, nonenveloped viruses include adenoviruses and picornaviruses, such as rhinovirus and encephalomyocarditis virus (EMCV). A range of virus inactivation methods exist that can reduce the likelihood of survival or transmission of viruses via direct contact or fomites by disrupting the lipid membrane of enveloped viruses (10). Such virucidal products include those targeted for personal hygiene use, such as soaps or hand gels that can be targeted to high-touch surfaces like the hands (10, 11). Additional measures are those that target the environment, such as surface disinfectants (12–14).

During a pandemic, there is likely to be an increased demand for products that eliminate viral infectivity from surfaces. Coniferous trees and some other plants produce liquid resin, which seals wounds in tree bark and protects the plant against pathogens and herbivores. Coniferous rosin contains resin acids, such as abietic acid and dehydroabietic acid, which are lipid-soluble diterpenoid carboxylic acids (15). Resin acids have been shown to have antibacterial properties, especially against Gram-positive bacteria (16, 17). Rosin can be collected from naturally occurring trees, but a commercially more important source of resin acids is crude tall oil, a side-stream of the cellulose processing industry. Here, we aimed to determine whether rosin soap exhibited virucidal activity against clinically relevant pathogenic human viruses. The viruses used in this study include the enveloped viruses IAV, respiratory syncytial virus (RSV), SARS-CoV-2, and EMCV. Initially, 2.5% rosin soap was evaluated for its virucidal activity for all enveloped viruses examined using liquid phase assays under standardized laboratory conditions. Here, we demonstrate the potent virucidal activity of rosin soap against pathogenic enveloped viruses, supporting its further development as a surface disinfectant.

## RESULTS

### Rosin soap reduces the infectivity of influenza virus.

**FIG 1:**
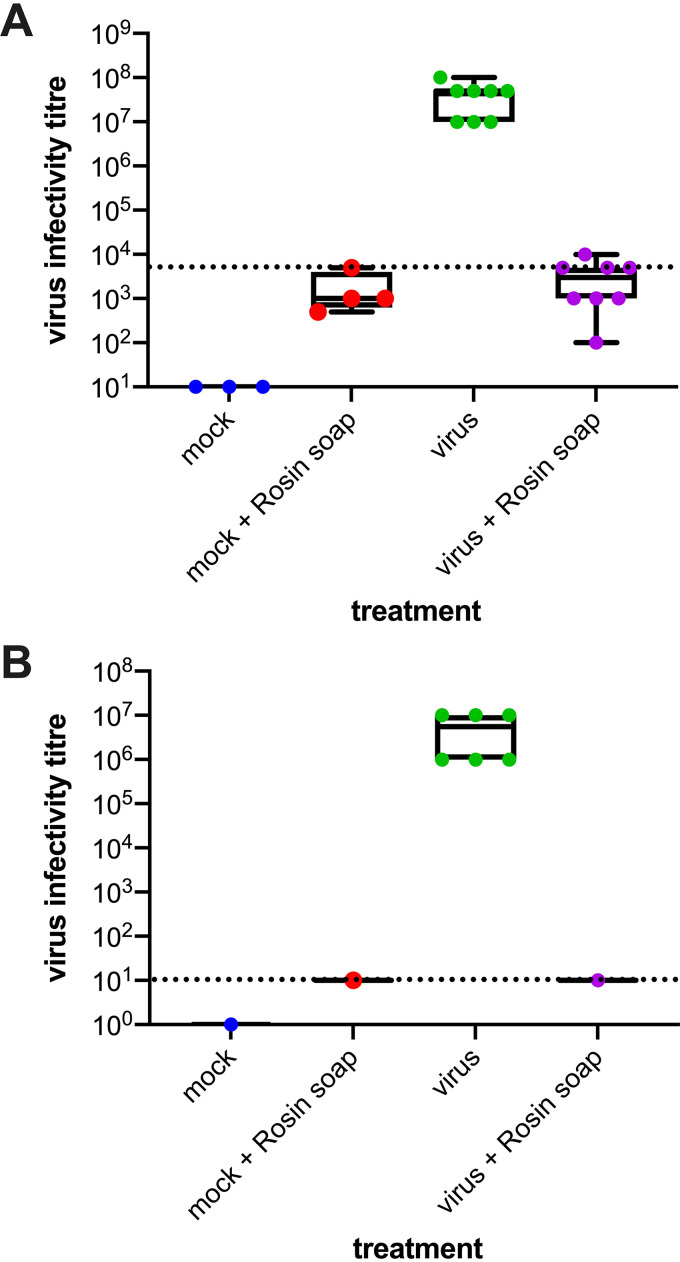
Effect of rosin soap treatment on IAV (strain WSN) infectivity in solution compared to mock DMEM without (A) and with (B) the removal of residual rosin acid by filtration. IAV suspension was incubated with rosin soap solution at 37°C for 5 min before the residual infectivity was determined via dilution on susceptible cells (MDCK cells). The infectious virus titer corresponds to the reciprocal of the final dilution showing a virus-induced cytopathic effect. The dashed line delineates the dilution at which the rosin soap treatment was toxic to the MDCK cells.

Novel solutions for disrupting the transmission of human pathogens are required. Given that it exhibits antibacterial activity, we hypothesized that rosin soap may inhibit the transmission of pathogenic human viruses by killing the viruses on surfaces. We thus took rosin soap and assessed whether it could reduce the infectivity of IAV (strain WSN). We chose IAV because it is a model enveloped human RNA virus and is a significant human pathogen. Furthermore, IAV achieves high titers during propagation in cell culture and is highly cytopathic (rapid cell death and rounding) in traditionally used cell lines such as Madin-Darby canine kidney (MDCK) cells, which together allow the facile and sensitive determination of high levels of inhibition to viral infectivity. To determine whether rosin soap powder could reduce the infectivity of IAV, we incubated influenza virus stocks with rosin acid (2.5% [wt/vol]) at 37°C for 30 min and measured the residual infectivity using a limiting dilution assay, assessing the cytopathic effect 72 h later, in comparison to virus- and Dulbecco’s modified Eagle medium (DMEM)-only controls. In these initial experiments, incubation of IAV with rosin soap powder gave at least a 10,000-fold reduction in infectivity (Fig. 1).

Precluding a precise determination of the reduction in infectivity is the relatively high limit of detection in this assay, due to the cytotoxic effect of residual rosin soap on cell viability, which is necessary for the detection of IAV infectivity. Throughout our studies, we were hindered by the relatively high cytotoxicity of rosin acids at the maximum concentration on the cells used to measure residual viral infection. This relatively high limit of detection prohibited us from determining whether there existed any viral infectivity remaining. To decrease the cytopathic effect and thus reduce the background, we filter-purified our virus/rosin acid preparations prior to infectivity measurements. The experimental conditions were room temperature (RT) for 5 min. These experiments demonstrated a removal of the background cytotoxicity and the lower limit of detection: Enhanced virucidal activity against IAV (1,000,000-fold) was observed (i.e., only 0.00001% remaining). These data suggest that rosin soap very likely can inactivate all infectious virus particles in each sample at 2.5% (wt/vol), although we cannot formally prove this.

### Assessment of the virucidal breadth of rosin soap.

**FIG 2:**
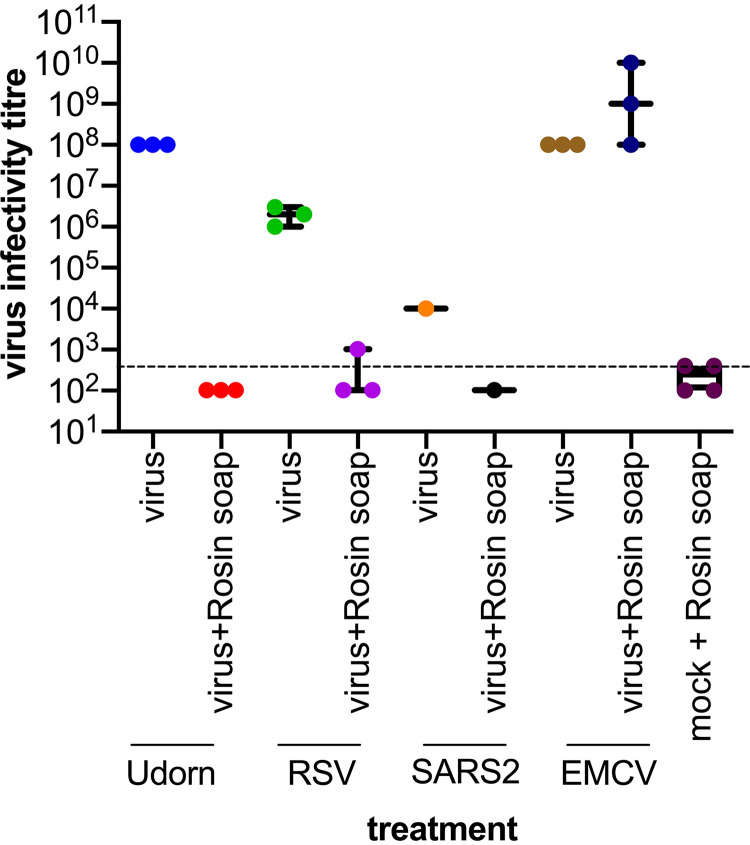
Effect of rosin soap treatment on a panel of virus infectivity in solution compared to mock DMEM. Three enveloped (IAV strain Udorn, RSV, and SARS-CoV-2 [SARS2]) and one nonenveloped EMCV virus were used. Virus suspensions were incubated with rosin acid solution at 37°C for 5 min before the residual infectivity was determined via dilution on susceptible cells (MDCK cells for IAV; Vero cells for RSV, SARS2, and EMCV). The infectious virus titer corresponds to the reciprocal of the final dilution giving a virus-induced cytopathic effect. The dashed line delineates the dilution at which the rosin soap treatment was toxic to the susceptible cells.

Given its effect on IAV infectivity, we hypothesized that rosin soap may also inhibit other viruses. To this end, we investigated the virucidal activity of rosin soap against another IAV strain (the H3N2 strain Udorn), RSV, and SARS-CoV-2, as well as the nonenveloped EMCV. RSV and SARS-CoV-2 are representatives from two groups of viruses, the pneumoviruses and the coronaviruses, and are themselves significant human pathogens. EMCV is a model nonenveloped virus and a pathogen of pigs and other mammals, such as nonhuman primates. We carried out the same protocol as used above for IAV WSN and measured the residual viral infectivity using virus-specific means. The conditions for these experiments were room temperature for 5 min. In this series of experiments, all enveloped viruses were inhibited by rosin soap, although to different degrees, demonstrating that the activity of rosin soap is not limited to WSN or IAV (Fig. 2). In all cases, treatment with rosin soap brought the infectivity down to baseline, and the fold inactivation was thus highly dependent on the starting concentration (e.g., greatest for Udorn and lowest for SARS-CoV-2). However, essentially all infectivity was brought to below the limit of detection, which is highly suggestive of nearly complete inhibition of infectivity (see previous experiment). Interestingly, neither rosin soap nor Triton X (data not shown) inhibited the nonenveloped EMCV. The susceptibility of enveloped viruses (and not the nonenveloped virus) to rosin acids suggests that the viral lipid membrane is a major target of inactivation.

### Virucidal activity of rosin soap is dependent on concentration.

**FIG 3:**
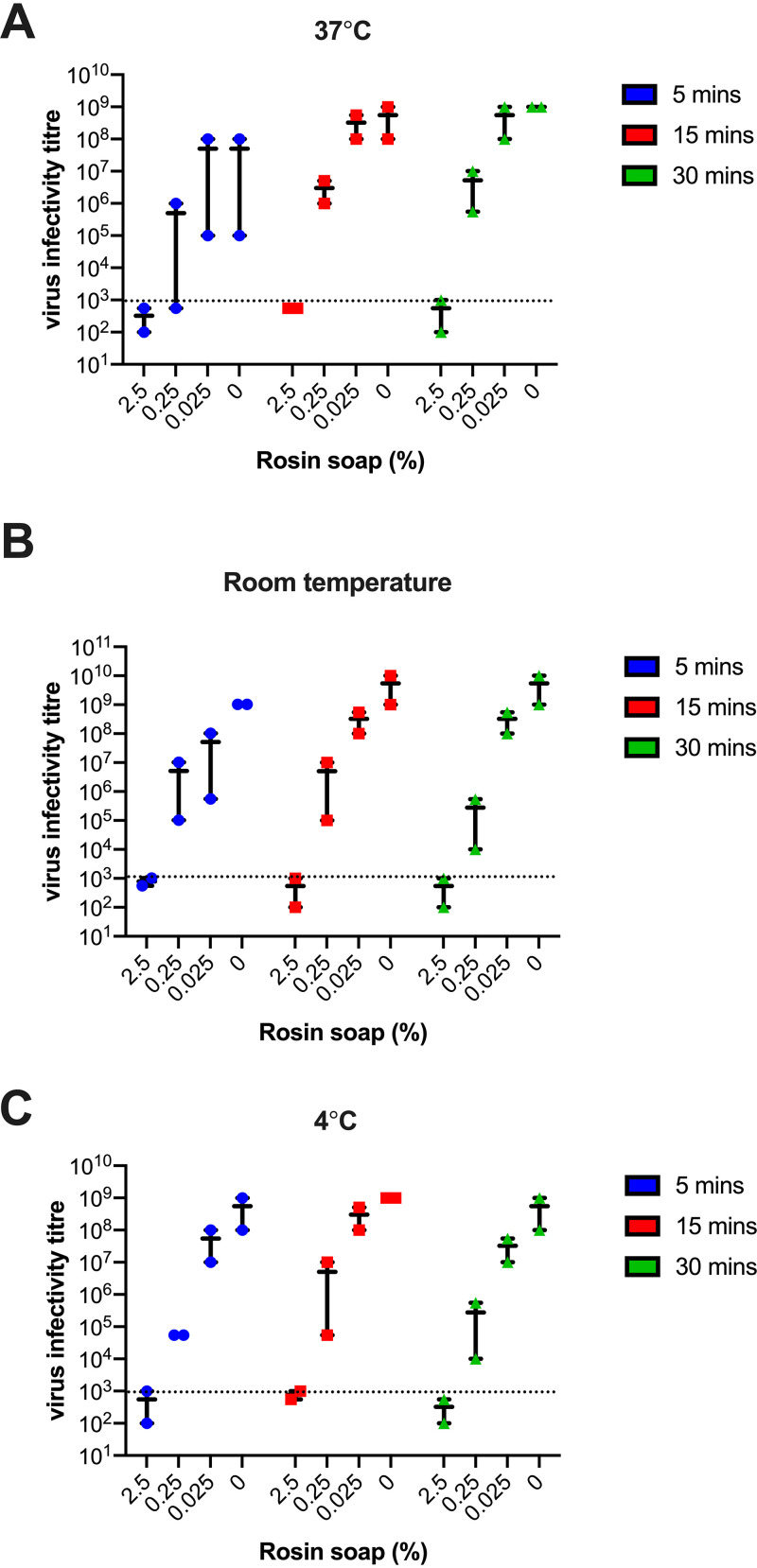
Effect of rosin soap treatment on IAV infectivity in solution compared to mock DMEM at different concentrations, treatment times, and temperatures. IAV suspensions were incubated with rosin soap solution (final concentration: 2.5, 0.25, or 0.025% [wt/vol]) under distinct conditions before the residual infectivity was determined via dilution on susceptible cells (MDCK). The effect of temperature—37°C (A), room temperature (B), or 4°C (C)—is shown alongside the incubation time—5 (blue), 15 (red), or 30 min (green). The infectious virus titer corresponds to the reciprocal of the final dilution showing a virus-induced cytopathic effect. These experiments were carried out in three replicates in two independent experiments.

To understand more about the physiochemical dependence of the potent activity exhibited by rosin soap against enveloped viruses like IAV, RSV, and SARS-CoV-2, we next determined the effect of the rosin soap concentration, temperature, and incubation time on its virucidal activity. All previous experiments were carried out with a concentration of 2.5% (wt/vol), a time of 5 min, and at room temperature, so here, we decided to alter the concentrations (2.5, 0.25, and 0.025% [wt/vol]), together with the incubation times (5, 15, and 30 min) and incubation temperatures (37°C, room temperature, and 4°C). Across all experiments, the virucidal activity of rosin soap was only dependent on the concentration, with 2.5% showing seemingly complete activity against IAV and a reduction in inhibition observed for each reduction in concentration (Fig. 3A). In contrast, the virucidal activity was independent of the incubation temperature (4°C, room temperature, and 37°C) and incubation time, with there being little difference between a 5-min incubation compared to a 30-minute incubation (Fig. 3A to C). These data demonstrate the rapid and efficacious activity of rosin acids against the enveloped virus IAV only when a critical concentration threshold has been reached.

## DISCUSSION

Infectious pathogenic human viruses, including SARS-CoV-2, can persist in the environment for extended periods of time, facilitating transmission via direct contact and/or through environmental contamination (18). Strategies for eliminating such infectivity from inanimate and animate surfaces is required. The exploration of strategies that are of natural origin is warranted. To this end, we sought to investigate whether rosin soap has antiviral activity, due to its reported antibacterial activity (16). Our work, presented here, shows that rosin soap exhibited rapid and potent virucidal activity against pathogenic human enveloped viruses but was not effective against a prototypic nonenveloped virus, EMCV.

Critically, we investigated the limits of the virucidal activity of rosin soap by altering the temperature, concentration, and time of incubation. Using IAV as a model enveloped virus, this virucidal effect was dependent on the concentration of the product rather than the incubation temperature or time. Interestingly, while the virucidal activity of rosin soap was not influenced by the length of exposure (5 to 30 min) or incubation temperature (4°C, room temperature, and 37°C), the only factor that did influence the virucidal activity was the rosin soap concentration, with 2.5% (wt/vol) being the most effective. Higher concentrations of rosin soap led to the rapid and potent loss of infectivity of IAV and other enveloped viruses. The fact that neither temperature nor time had a major impact on the efficacy suggests that this product has highly potent virucidal activity.

Mechanistically, our results showing the lack of efficacy against nonenveloped viruses suggests that the target for the antiviral activity of rosin soap is the viral envelope, which is composed of a phospholipid bilayer. The virus envelope is critically required for infectivity, facilitating protection of genomic material and facile entry (catalyzed by viral fusion protein machinery) into target host cells via virion-to-cell membrane fusion, either at the plasma membrane or endosomal compartment membranes (19). Loss of virion envelope integrity will prevent the entry and release of infectious virus genomes into host cells, which is likely responsible for the virucidal activity observed herein. How rosin soap might disrupt the envelope is unknown, but rosin soaps likely act as surfactants; further studies are required to determine this. Precisely how rosin soap impacts the viral envelope is not known at this stage. Rosin soap is a mix of products, and it would be useful to look at the individual compounds—both in terms of the resin acids and the carboxylic acids—as has been done for other virucidal products (20). However, there is limited commercial availability of these, and they also have limited solubility in pure solution.

Unfortunately, due to the cytotoxic nature of rosin soap at high concentrations (from 0.25% to ∼0.0025%) in our *in vitro* cell culture conditions, we were not able to completely negate this background toxicity in our virus infectivity assays (which rely upon cellular integrity), even following the purification of our virus/soap mixes by filtration. However, our data suggest that it is highly likely that rosin soap inactivates the vast majority of infectious particles in a given preparation. Using IAV, which grows to very high titers, we were able to demonstrate nearly complete inactivation. It is worth noting that the level of virus titer used in these experiments is higher than that likely present in most “real world” scenarios/environments (21). Despite our observation of toxicity in cell culture conditions, rosin salves have been found to be safe and effective in wound care (22).

The virucidal activity of rosin soap when viruses are dried onto surfaces is an area that needs further research, as viruses such as SARS-CoV-2 persist on surfaces and are a source of infection transmission (23). This would determine if rosin soap can be formulated into products that could be used as a commercial surface disinfectant for premises including hospitals. A wider variety of viruses could also be examined to determine if rosin soap exhibits the same virucidal activity against most or all enveloped viruses, such as SARS-CoV-2. Rosin soap did not inhibit the nonenveloped virus, EMCV. Other nonenveloped viruses, such as rhinoviruses or noroviruses, could be examined to determine if it is only EMCV that rosin soap does not inhibit.

In conclusion, we demonstrated the virucidal activity of rosin soap against multiple pathogenic human enveloped viruses.

## MATERIALS AND METHODS

### Cell culture.

Mammalian cell lines (MDCK [Madin-Darby canine kidney] and Vero [African green monkey] cells) were cultured in DMEM (high glucose) supplemented with fetal bovine serum (5% [vol/vol]) and penicillin/streptomycin (1% [vol/vol]). The cell cultures were maintained in flasks (175 cm^2^) and passaged routinely.

### Viruses.

Stocks of the representative viral strains, including influenza A virus (Udorn, WSN), respiratory syncytial virus (RSV-A2), SARS-CoV-2, and EMCV, were prepared using standard virology techniques on Vero (SARS-CoV-2 and EMCV), MDCK (influenza A virus), and Hep-2 (RSV) cells. For culture of IAV Udorn, serum-free medium was used, supplemented with TPCK (tosylsulfonyl phenylalanyl chloromethyl ketone)-treated trypsin (Sigma-Aldrich) at a concentration of 1 μg/mL. The infectious stocks were produced and titrated in their respective cell lines before use in the virucidal activity experiments. All virus work was carried out in the biological safety level 2 (BSL2) or BSL3 (SARS-CoV-2) facilities at QUB.

### Tall oil.

The rosin soap was produced from crude tall oil by Forchem Ltd. (Rauma, Finland). It was a water solution obtained from dried rosin salt, consisting of less than 10% sodium salts of tall oil fatty acids and over 90% sodium salts of resin acids. The resin acids and fatty acids of the product originated from the coniferous trees Pinus sylvestris L. and Picea abies L. The most abundant resin acid types included abietic acid, dehydroabietic acid, pimaric acid, and palustris acid. The pH of the raw undiluted product was pH 8 to 9, while diluted in DMEM at an antiviral concentration, it was pH 7 to 7.5, as estimated using pH indicator strips.

### Inactivation protocol.

The virus inactivation assays were carried out in 96-well plates. Initially, complete DMEM (100 μL) was added to each well, except the first column, which was used to incubate the virus and product. Three concentrations of rosin soap powder were tested under each condition in duplicate (2.5%, 0.25%, and 0.025% [wt/vol]). Rosin soap (Forchem Ltd., Rauma, Finland) was dissolved in double-distilled water (ddH_2_O). The negative control contained no virus and was incubated at 37°C. To each well of the first column, 100 μL of treatment and 100 μL of virus was added. After exposure to the experimental conditions, which were time (5, 15, or 10 min) and temperature (4°C, room temperature, or 37°C), 10-fold serial dilutions were carried out. Following dilution of the virus, permissive cells were added (100 μL) and incubated for 2 to 3 days. Viral infectivity was measured as the reciprocal of the final dilution showing a cytopathic effect, following manual investigation with a light microscope.

### Filtration.

To remove residual rosin soap from the treated virus inoculum and hence lower the level of cytotoxicity of the treatment when measuring infectivity of virus preparations, Amicon Ultra-15 centrifugal filter units (Merck) were used. One hundred microliters of virus (WSN) was added to 100 μL of rosin soap (2.5% [wt/vol]) for 5 min at room temperature. The 200 μL (WSN/rosin soap powder) was washed through the filter units four times with 12 mL fresh DMEM (supplemented with fetal bovine serum, 5% [vol/vol], and penicillin/streptomycin, 1% [vol/vol]). Tenfold serial dilutions were carried out. Following dilution of the virus, permissive cells were added (100 μL) and incubated for between 2 and 3 days. Viral infectivity was measured as the reciprocal of the final dilution showing a cytopathic effect.
